# The use of drug-induced sleep endoscopy in England and Belgium

**DOI:** 10.1007/s00405-018-4939-y

**Published:** 2018-03-19

**Authors:** Vik Veer, Henry Zhang, Jolien Beyers, Olivier Vanderveken, Bhik Kotecha

**Affiliations:** 1 Royal National Throat Nose and Ear Hospital, 330 Gray’s Inn Rd, London, WC1X 8DA UK; 2 Queens Hospital, Rom Valley Way, Romford, RM7 0AG UK; 3 Department of ENT, Head and Neck Surgery, Antwerp University Hospital, Edegem, and, Faculty of Medicine and Health Sciences, University of Antwerp, Antwerp, Belgium

**Keywords:** DISE, Drug-induced sleep endoscopy, Obstructive level, OSA, Obstructive sleep apnoea, Sleep, Survey

## Abstract

**Purpose:**

The purpose of this international survey is to ascertain the current practice of drug-induced sleep endoscopy (DISE) for patients with sleep-disordered breathing (SDB) by Otolaryngologists in the United Kingdom and Belgium. We compare the results with recommendations from the European Position Paper on drug-induced sleep endoscopy.

**Methods:**

An online questionnaire was circulated to Consultant Otolaryngologists, independent practitioners, and trainees across the two countries. Eleven questions were used in total.

**Results:**

181 responses from the UK and 117 responses from Belgium were received, mostly from consultants and independent practitioners. SDB was a common presentation to ENT practice, seen by over 90% of clinicians. The use of DISE varied greatly between the two countries (72.9% Belgium, 26.1% UK). 54.1% of Belgian respondents use DISE on over 50% of their patients, compared to only 32.4% of British clinicians. Attitudes of surgeons towards the diagnostic value of DISE varied; in Belgium, the majority (54%) gave a rating of 3 or more (1 = useless to 5 = essential), with no respondents giving a score of 0 (useless). In the UK only 16% of respondents felt DISE had useful clinical value, with 25 respondents deeming it ’useless’. The majority opt for DISE when non-surgical therapies fail (51.4% UK, 61.3% Belgium). The majority of participants do not use objective measures for depth of sedation (75.7% UK, 66.7% Belgium), with a marked variation on anaesthetic methods. 62.2% of UK clinicians do not use a classification system, whereas in Belgium the majority of clinicians (60.8%) use the VOTE grading system.

**Conclusions:**

Clinicians in Belgium were more favourable to using DISE than in the UK. Differences in its clinical effectiveness were apparent between the two countries. A consensus on patient selection, method of sedation and an effective classification system seemed to be lacking from both countries. Further education is required to raise awareness for the use of DISE.

## Introduction

Sleep-disordered breathing (SDB), describes a spectrum of pathologies from ‘simple snoring’ to obstructive sleep apnoea (OSA). Untreated OSA can lead to diminished quality of life, cardiovascular disease, stroke and motor vehicle accidents. Therefore, adequate treatment is of utmost importance [1, 2]. Although continuous positive airway pressure (CPAP) is the gold standard treatment for OSA [3], 50% of patients permanently abandon CPAP in the first week of use. 12–25% of the remaining users abandon CPAP use in the following 3 years, leading to an estimated overall rate of non-adherence as high as 83% [4]. For this reason, alternative therapies for OSA are often sought. Drug-induced sleep endoscopy (DISE), otherwise known as sleep nasendoscopy, is an investigation that attempts to delineate the level of upper airway obstruction, allowing a targeted treatment for SDB. DISE utilises a light anaesthetic sedation to provide a state similar to natural sleep so that flexible nasendoscopy may be performed and the SDB obstructive level, direction and degree may be identified within the collapsible segment of the individual OSA patient’s upper airway. The use of sleep endoscopy began in 1978 when Borowiecki performed an endoscopic examination of the upper airway (UA) during natural physiological sleep [5]. The limitations of relying on spontaneous sleep with lack of resources and time made this procedure difficult. In 1991, Croft and Pringle reported on patients undergoing UA assessment for OSA under sedation [6]. This enabled the procedure to be carried out routinely during the day, as well as better tolerance of the endoscope. DISE has since become increasingly popular worldwide. The European Position Paper from 2014 recommends DISE in patients with socially disturbing snoring and OSAHS, in whom non-CPAP therapy is being considered [7]. Furthermore, DISE may be performed on patients who have failed or are having difficulty in tolerating CPAP. In addition, patients who have failed previous surgery may benefit from DISE, as information could be provided to recommend oral appliance therapy (OAT) or further surgery to address relevant anatomical cause of obstruction [8]. However, DISE remains a controversial topic, particularly in the UK. Criticisms of the validity of DISE focus on whether a light sedation is a clinically accurate representation of sleep, and if the results of DISE affect treatment choices and patient outcomes.

The primary aim of this study was to ascertain the use of DISE and clinicians’ opinions about its validity. A national survey was developed for English and Belgian surgeons to investigate these aims, and also to identify any geographic variations in these two countries. The supposition was that there would be variations in the use of DISE and possibly alternative DISE techniques were being employed. Secondarily, the authors sought to determine the range of perceptions and opinions of DISE’s effectiveness in the investigation of SDB. The authors were keen to compare and contrast these variables between the two countries.

## Methods

The online questionnaire developed by the authors is provided in “Appendix 1”. This was disseminated online to the ENTUK membership on the 4th August 2015 and to the members of the Royal Belgian Society of Otorhinolaryngology and Head and Neck Surgery on the 3rd May 2016 with a reminder email on the 30th May 2016 using the SurveyMonkey online software and the Qualtrics Survey Software, respectively. Eleven questions were used; however, depending on answers provided several elements of the survey could be skipped, particularly if SDB patients were not seen as a part of the participant’s medical practice. Circumventing further questions was also possible if DISE was not used in the normal investigation of these patients. Geographic location and DISE technique employed were examined and opinions of participants were sought. Results were collected anonymously and a translated version of the survey was distributed to the Belgian surgeons in the Dutch, the French and the English language.

## Results

In Britain the email inviting surgeons to complete the survey was sent to 1354 contacts on two occasions 2 weeks apart. Five hundred and fifteen people opened the email on the first occasion, and 375 opened the email on the second attempt. A total of 181 people completed the survey in total, making a response rate of 13.4%.

The Belgium survey had a superior response rate with an email sent to 450 contacts and 117 completing the survey (26.0%). The results of the questionnaire are presented in Tables 1, 2, 3, 4, 5, 6, 7, 8. Please note some respondents gave multiple answers.

**Table 1:** Question and results

Question 1. In your normal clinical practice (NHS or private), do you see patients presenting with SDB?		
	British responses (%)	Belgian responses (%)
No	18 (9.9%)	5 (4.3%)
Yes	163 (90.1%)	112 (95.7%)
Total	181	117

*NHS* National Health Service System, *SDB* sleep disordered breathing

**Table 2:** Methods of upper airway obstruction analysis

Question 2. In patients with SDB, which methods do you use to determine the level of upper airway obstruction? (tick all that may apply)		
	British responses (%)	Belgian responses (%)
Awake flexible nasendoscopy examination	138 (87.9%)	99 (92.5%)
Muller’s manoeuvre (with awake flexible nasendoscopy)	92 (58.6%)	71 (66.4%)
Simulated snoring (with or without awake flexible nasendoscopy)	32 (20.4%)	49 (45.8%)
Apneagraph	17 (10.8%)	5 (4.7%)
Other	20 (12.7%)	21 (19.6%)
Total	157	107

**Table 3:** Utilisation of DISE for assessment of patients with SDB

Question 3. Do you use DISE (drug-induced sleep endoscopy) to assess your SDB patients?		
	British responses (%)	Belgian responses (%)
No	116 (73.9%)	29 (27.1%)
Yes	41 (26.1%)	78 (72.9%)
Total	157	107

**Table 4:** Percentage of patients with SDB investigated with DISE

Question 4. Approximately, what percentage of your SDB patients do you investigate with DISE?		
	British responses (%)	Belgian responses (%)
Less than 25%	13 (35.1%)	13 (20.6%)
25% to less than 50%	12 (32.4%)	16 (25.4%)
50% to less than 75%	4 (10.8%)	14 (22.4%)
75% or greater	8 (21.6%)	20 (31.7%)
Total	37	63

**Table 5:** Selection criteria of patients with SDB for investigation with DISE

Question 5. Which of the following best represents your criteria for selecting SDB patients for investigation with DISE?		
	British responses (%)	Belgian responses (%)
All patients that present with symptoms of SDB	6 (16.2%)	15 (24.2%)
Patients in whom examination in clinic does not identify an obvious cause for their SDB symptoms	11 (29.7%)	27 (43.5%)
SDB patients who have failed or are intolerant of non-surgical therapies (nasal splints, mandibular advancement splints, home CPAP)	19 (51.4%)	38 (61.3%)
Other	13 (35.1%)	22 (35.5%)
Total	37	62

**Table 6:** Anaesthetic choice for induction of snoring state during DISE

Question 6. By which method does your anaesthetist induce the snoring state during DISE?		
	British responses (%)	Belgian responses (%)
Boluses of propofol only	10 (27.0%)	16 (27.0%)
Boluses of propofol with midazolam	18 (48.6%)	8 (13.0%)
A computerised target concentration-controlled infusion (TCI), of proprofol	3 (8.1%)	26 (43.0%)
Midazolam only	2 (5.4%)	0
Other	4 (10.8%)	10 (17%)
Total	37	60

**Table 7:** Objective measures employed to gauge depth of sedation during DISE

Question 7. What objective measures do you employ to gauge the depth of sedation during DISE?		
	British responses (%)	Belgian responses (%)
None	28 (75.7%)	44 (66.7%)
Bispectral index monitoring (BIS)	6 (16.2%)	12 (18.2%)
Electroencephalography (EEG)	1 (2.7%)	4 (6.1%)
Other	5 (13.5%)	6 (9.1%)
Total	37	60

**Table 8:** Classification system used during DISE

Question 8. Which classification/grading system do you use? (tick all that apply)		
	British responses (%)	Belgian responses (%)
None—I describe what I find	23 (62.2%)	9 (12.1%)
VOTE	0 (0%)	45 (60.8%)
Nose Oropharynx Hypopharynx and Larynx (NOHL)	4 (10.8%)	10 (13.5%)
Croft & Pringle	11 (29.7%)	3 (4.1%)
Other	5 (13.5%)	7 (9.5%)
Total	37	61

To limit the number of questions that participants needed to answer, this question was used to divert those participants who do not see patients presenting with conditions that DISE is designed to investigate. These participants were transferred to question 9, to gain an understanding of their perceptions of DISE, even though they do not use it.

The original questionnaire was designed to include both question 2 and 3 as one, but due to a limitation in the SurveyMonkey software, transferring participants who answered question 3 negatively was not possible if there were other answers available. The decision was made, therefore, to split the question in two. There were two other valid responses provided as examples of other methods of determining the level of obstruction. One British participant suggested ‘lateral radiography’ presumably for identification of an adenoidal pad in paediatric patients unable to tolerate awake nasendoscopy. Three Belgian participants mentioned ‘Imaging’ with no further information. There have been studies which use imaging such as CT scan during wake in supine position using computational fluid dynamics [9] and other reports on MRI during natural sleep [10], which is possibly what these three participants are referring to. Sleep studies and other similar answers were rejected, as they do not provide an indication of the level of obstruction. The respondents were in question 2 permitted to choose multiple answers.

Question 3 showed a clear difference between the two countries as approximately 74% in Britain stated that they did not use DISE in their practice, whereas about 73% of Belgian respondents stated that they do. Participants who answered negatively at this point were transferred to question 9 to ascertain their opinions of DISE.

There was again a significant trend by the Belgian respondents to state that they use DISE, compared to the British survey results.

The respondents were in this question permitted to choose multiple answers explaining how the total numbers are so low. All of the Belgian answers for the category stated that they would use DISE in patients who did not meet the CPAP criteria for treatment. Three respondents in Britain had similar answers to the Belgians, but two used DISE for children who did not improve after adenotonsillectomy. The rest of the British responses were variations on the theme of using DISE for only those who met local BMI and AHI criteria, and also wanted surgery.

It is difficult to draw clear conclusions from this question as there has been such a discrepancy in the previous two questions that this would have a difficult to interpret impact on these answers.

The obvious difference between the two countries here was the main method of induction by percentage. We believe this is likely to be due to the training differences between the two countries and the preferences of the senior authors and their anaesthetists. In the ‘other’ bracket the responses were similar between the countries in that one person each used midazolam with a TCI of propofol. Other responses were simply that the surgeons did not know the technique used by their anaesthetists.

The responses here were relatively similar between the two countries. The difficulty of measuring depth of sedation is highlighted here, as well as clinicians’ reluctance to accept Bispectral index monitoring (BIS), either due to cost, or lack of knowledge of the algorithm that is used to produce the output value. One British respondent stated that they did not find BIS useful and, therefore, its use was abandoned.

The classification systems employed by the two countries varied greatly. The most common classification system used among the respondents of the British survey was the Croft & Pringle system [6] (29.7%); however, three of the people who had picked ‘other’ stated that they used the royal national throat nose and ear (RNTNE) hospital system which is actually Croft & Pringle’s system. 10.8% use the nose oropharynx hypopharynx and larynx (NOHL) classification [11], but no one picked the VOTE classification [12] The majority of British respondents (62.2%) stated that they do not use a specific classification opting instead to describe what they observe during DISE. In the Belgian survey, the most common classification system used was VOTE (60.8%), followed by NOHL (13.5%). Only 4.1% used Croft & Pringle, with 9.5% of respondents stating ‘other’. 12.1% of Belgian respondents prefer to describe what they observe.

Question 9. In your opinion, how would you rate the diagnostic value of DISE in the investigation of SDB?

The answers were graded 0 point for ‘useless’ up to five points for ‘essential’. A significant variation was seen between UK and Belgian responses. In the UK, 149 respondents averaged a score of 2.95. In Belgium 90 clinicians answered the question, averaging a score of 4.6. Forty seven UK respondents chose to share their opinions on DISE and these were separated into the following broad categories. Fourteen stated that they did not find DISE to be of benefit when it was used, and ten stated that DISE is not true sleep and, therefore, not a valid measure. Closely allied to this, four respondents stated that there is no evidence for this investigation, and six felt it was either time consuming or too expensive. Eight stated that they either had no experience or logistically were unable to perform DISE, and one gave the opinion that DISE should not be performed in an NHS setting. Four felt that DISE was only useful in a selective number of patients, whereas two simply stated that DISE was a useful investigation with a further three stating that results are dependent on anaesthetic support.

Question 10. What percentage of your SDB patients do you see in the private sector compared to in the NHS?

**Fig. 1:**
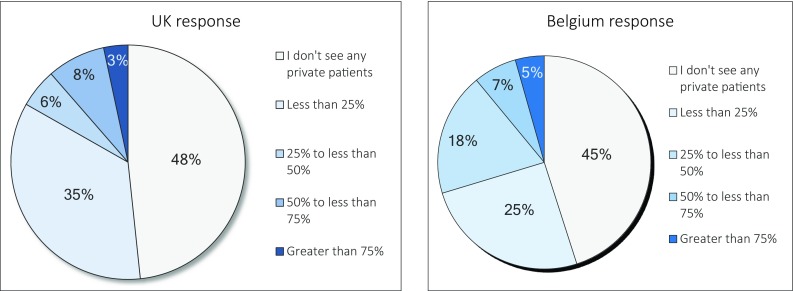
Percentage of patients seen in the public and private sector

The responses to this question are provided in Fig. 1.

Question 11. What is your current grade? Please pick a grade which most closely represents your current status.

This question showed that the majority of respondents to this survey were independent practitioners (86% of UK and 77% of Belgian respondents were either consultants or associate specialists).

## Discussion

The treatment of OSA using CPAP varies according to patient acceptance, tolerance, and compliance [13]. Alternatives to CPAP such as oral appliance therapy (OAT) and surgery require careful diagnosis, to ascertain the level of upper airway (UA) collapse. Although surgical treatment has, in the past, been of varying degrees of success, thorough assessment of the level, degree and pattern of obstruction would lead to better selection of surgical techniques [14, 15]. Assessment of these patients requires knowledge of upper airway anatomy and function, as well as the physiology of normal sleep, to determine the complexity of pharyngeal collapse. Due to changes in muscle tone during sleep, observation of the upper airway in awake patients might be of limited value [16]. In our survey an overwhelming majority of respondents from both countries use awake fibreoptic endoscopic evaluation to assess the level of obstruction.

Only 26.1% of UK respondents from our survey use DISE to assess patients with SDB, compared to 72.9% of Belgian clinicians. Of these, the majority (51.4% of British and 61.3% of Belgian respondents) were for SDB patients who had failed or are intolerant of non-surgical therapies (nasal splints, mandibular advancement splints, home CPAP).

Possible explanation for the UK clinicians not utilising DISE may be due to the lack of funding and approval in the NHS setting. Treatment for snoring in terms of surgery is included in the “procedures of limited clinical effectiveness (POLCE)” [17, 18]. Furthermore, 85% of the British physicians mentioned they see most of their SDB patients in the private sector, compared to 70% of the Belgian physicians.

The results of this survey point out that the ulitisation of DISE in clinical ENT practice in Belgium is rather well implemented. A majority of Belgian ENT surgeon (61.3%) do use DISE in their routine clinical evaluation of OSA patients that would be candidates for non-CPAP therapy, with most of them using a classification system. The most popular scoring system for DISE among ENT surgeons in Belgium turned out to be the VOTE grading system.

Another explanation for the difference between both countries can be found in the results of another question asked in our survey. In the UK, physicians gave a mean score of 2.95 on a scale of 5 when they were asked if they thought DISE is useful in the investigation of SDB, compared to a score of 4.6 in the Belgian population. One possible explanation of this may be differences in training programmes between the two countries.

### Methods of assessment during DISE

Our survey showed great heterogeneity in both countries in the method of sedation, varying from boluses of propofol (27.0% in both countries) to combination with midazolam (48.6% in the UK versus 13.0% in Belgium) to a computerised target concentration-controlled infusion (TCI) of propofol (8.1% in the UK versus 43.0% in Belgium). There is evidence showing that UA findings do not differ using propofol or midazolam [19], with the preferred option being TCI [20]. Irrespective of delivery method, safety of sedation is paramount, and the need to mimic natural sleep is important to aid accurate diagnosis. EEG-derived indices such as Bispectral (BIS) monitoring, which assess depth of sedation, should be used if available [21, 22], BIS levels of 50–70 are recommended. The survey showed that in both the UK and Belgium, the majority of clinicians do not use any monitoring during DISE to assess the depth of sedation (75.7% in the UK and 66.7% in Belgium), only 16.2% of UK and 18.2% of Belgian respondents use BIS, with a small minority (16.2% UK, 15.2% Belgium) using other methods such as EEG. Regardless of monitoring method, there was no consensus in Britain on a definitive classification method, most surgeons describing what they find during DISE (62.2%). Although the VOTE classification system was used by a majority of Belgian respondents (60.8%), a variation was still apparent. Furthermore, although the VOTE system does give good inter-rater agreement, it has been criticised for being over-simplified [7]. Finally, the majority of the respondents to this survey were independent practitioners in the UK and Belgium (86 and 77% respectively). In the literature, the validity and reliability of DISE in experienced observers have been demonstrated [23], but assessment remains subjective and differences in clinical experience may cause some variations [23–25]. Furthermore, it was earlier described that for some specific collapse patterns, differences in scoring by the experienced observers remain despite long-term use of the same uniform scoring system, when assessing the level, direction and degree of collapse [26].

## Conclusion

This survey demonstrates the broad range of opinions clinicians have of DISE, both in the UK and Belgium. SDB is a common clinical presentation in both countries. In the UK, a low opinion of the clinical effectiveness of DISE may have led to most clinicians not employing it in their practice. Patient selection for DISE varied greatly, and those that undertook the procedure had no consensus on sedation method, monitoring, or classification system. In Belgium, respondents were more positive about its use. A higher opinion of DISE’s usefulness may have led to a higher percentage of use in clinical practice. More concordant classification systems for interpretation and patient selection were present. In conclusion, the use of DISE for patients with SDB appears to be greatly underestimated by clinicians, mainly from the UK. The differences in opinions and practices by clinicians in the two countries could be due to geographical variation, as well as discrepancies in national healthcare provisions. That is, the National Health Service (NHS) in the UK does not fund all hospitals for using DISE as a diagnostic tool. Separate negotiations with the regional Primary care may be necessary in attaining funding [17, 18]. This could have an implication on training programmes for residents in the UK. Further education is warranted, to reach a consensus for its use and interpretation. More work is needed for a rigid, effective and simple to use system to determine the best treatment following DISE and as recently suggested by Vanderveken [27] there is a clear need to enhance the understanding and the uniformity when considering utilising DISE for evaluating upper airway obstruction.

## Appendix 1. The use of drug-induced sleep endoscopy. Questionnaire distributed to ENT surgeons in UK and Belgium

Question 1. In your normal clinical practice (NHS or private), do you see patients presenting with SDB?

Question 2. In patients with SDB, which methods do you use to determine the level of upper airway obstruction? (tick all that may apply)

**Table:**

Awake flexible nasendoscopy examination
Muller’s manoeuvre (with awake flexible nasendoscopy)
Simulated snoring (with or without awake flexible nasendoscopy)
Apneagraph
Other

Question 3. Do you use DISE (Drug Induced Sleep Endoscopy) to assess your SDB patients?

Question 4. Approximately, what percentage of your SDB patients do you investigate with DISE?

**Table:**

Less than 25%
25% to less than 50%
50% to less than 75%
75% or greater

Question 5. Which of the following best represents your criteria for selecting SDB patients for investigation with DISE?

**Table:**

All patients that present with symptoms of SDB
Patients in whom examination in clinic does not identify an obvious cause for their SDB symptoms
SDB patients who have failed or are intolerant of non-surgical therapies (nasal splints, mandibular advancement splints, home CPAP)
Other

Question 6. By which method does your anaesthetist induce the snoring state during DISE?

**Table:**

Boluses of propofol only
Boluses of propofol with midazolam
A computerised target concentration controlled infusion (TCI), of proprofol
Midazolam only
Other

Question 7. What objective measures do you employ to gauge the depth of sedation during DISE?

**Table:**

None
Bispectral index monitoring (BIS)
Electroencephalography (EEG)
Other

Question 8. Which classification/grading system do you use? (tick all that apply)

**Table:**

None—I describe what I find
VOTE
Nose Oropharynx Hypopharynx and Larynx (NOHL)
Croft & Pringle
Other

Question 10. What percentage of your SDB patients do you see in the private sector compared to in the NHS?

**Table:**

Less than 25%
25% to less than 50%
50% to less than 75%
75% or greater

Question 11. What is your current grade? Please pick a grade which most closely represents your current status.
